# Improved Efficiency of Cardiomyocyte-Like Cell Differentiation from Rat Adipose Tissue-Derived Mesenchymal Stem Cells with a Directed Differentiation Protocol

**DOI:** 10.1155/2019/8940365

**Published:** 2019-04-01

**Authors:** Blanca Rebeca Ibarra-Ibarra, Martha Franco, Araceli Paez, Elvira Varela López, Felipe Massó

**Affiliations:** ^1^Laboratory of Translational Medicine, UNAM-INCar Research Unit, Instituto Nacional de Cardiología, Ignacio Chávez, Mexico City, Mexico; ^2^Department of Nephrology, Instituto Nacional de Cardiología, Ignacio Chávez, Mexico City, Mexico

## Abstract

Cell-based therapy has become a resource for the treatment of cardiovascular diseases; however, there are some conundrums to achieve. *In vitro* cardiomyocyte generation could be a solution for scaling options in clinical applications. Variability on cardiac differentiation in previously reported studies from adipose tissue-derived mesenchymal stem cells (ASCs) and the lack of measuring of the cardiomyocyte differentiation efficiency motivate the present study. Here, we improved the ASC-derived cardiomyocyte-like cell differentiation efficiency with a directed cardiomyocyte differentiation protocol: BMP-4 + VEGF (days 0-4) followed by a methylcellulose-based medium with cytokines (IL-6 and IL-3) (days 5-21). Cultures treated with the directed cardiomyocyte differentiation protocol showed cardiac-like cells and “rosette-like structures” from day 7. The percentage of cardiac troponin T- (cTnT-) positive cells was evaluated by flow cytometry to assess the cardiomyocyte differentiation efficiency in a quantitative manner. ASCs treated with the directed cardiomyocyte differentiation protocol obtained a differentiation efficiency of up to 44.03% (39.96%±3.78) at day 15 without any enrichment step. Also, at day 21 we observed by immunofluorescence the positive expression of early, late, and cardiac maturation differentiation markers (Gata-4, cTnT, cardiac myosin heavy chain (MyH), and the sarcoplasmic/endoplasmic reticulum Ca^2+^ ATPase (SERCa2)) in cultures treated with the directed cardiomyocyte differentiation protocol. Unlike other protocols, the use of critical factors of embryonic cardiomyogenesis coupled with a methylcellulose-based medium containing previously reported cardiogenic cytokines (IL-6 and IL-3) seems to be favorable for *in vitro* cardiomyocyte generation. This novel efficient culture protocol makes ASC-derived cardiac differentiation more efficient. Further investigation is needed to identify an ASC-derived cardiomyocyte surface marker for cardiac enrichment.

## 1. Introduction

Stem cells are a source of immature renewable cells that can lead to the development of various cell types; this makes its use attractive for tissue regeneration. The differentiation capacity of the stem cells is well known; however, the differentiation efficiency is sometimes variable depending on the cell type and protocol used [1, 2]. Cardiomyocyte *in vitro* generation has advantages for clinical applications, controlling the number of cells, and knowing the cardiomyocyte subtype transplanted in patients with myocardial infarction [3, 4] or other cardiovascular diseases such as refractory angina or ischemic cardiomyopathy [5]. Great advances have been developed in this matter; nevertheless, there are some limitations to translate these findings to clinical applications [2].

Cardiomyocyte differentiation was described before in distinct types of stem cells such as mesenchymal stem cells (MSCs) [6, 7], embryonic stem cells (ESCs) [8, 9], and induced pluripotent stem cells (IPSCs) [1, 10, 11]. Despite having a high differentiation efficiency from ESCs and IPSCs, the use of these cells has been restricted in clinic usage because of their tumorigenic potential, dedifferentiation, and higher costs to generate them [2, 12]. Otherwise, MSCs such as adipose tissue-derived mesenchymal stem cells (ASCs) have shown a lower differentiation efficiency depending on the method used, but their lower tumorigenic potential, and costs, as well as easier accessibility, make them attractive to use for scale-up options and for clinical applications [4, 13].

Some reports have described the induction of ASC-derived cardiomyocyte-like cells with different approaches in different species (mouse, rat, rabbit, and human). Until now, there is no consensus on the best cardiomyocyte induction protocol. These strategies obtained a low and variable source of spontaneously beating cardiomyocyte-like cells sometimes expressing specific cardiac markers compatible with a cardiomyocyte morphology [6, 14, 15]. The great majority induce undifferentiated ASCs with a unique small molecule or growth factor [6, 7, 16–18]. Others have used cocultivated ASCs and cardiomyocytes, but its use is restricted for further scalability for clinical applications [15, 19]. Higher efficiency was observed by isolating the beating clusters; however, this method depends on the number of spontaneously beating cardiac-like cells [7]. In addition, very few studies have measured the differentiation efficiency towards cardiomyocytes from ASCs with a quantitative method that allows us to compare between different protocols and be able to identify which is optimal for further applications [7, 16].

Directed cardiomyocyte differentiation protocols consist in the manipulation of different signaling pathways via combination of some growth factors (BMP-4, VEGF, and bFGF), small molecules, and cytokines, among others, mimicking the embryonic cardiomyogenesis; as was observed in the recent years with ESCs and IPSCs, cardiomyocyte differentiation protocols achieve a higher differentiation efficiency (nearly 90%) with different kinds of combinations [1, 10, 11, 20–22]. So far, IPSC studies have overshadowed the studies carried out in ASCs, and very few studies have explored the use of directed cardiomyocyte differentiation protocols in ASCs [23].

Stem cell cardiac differentiation is a spatiotemporal complex process, and *in vitro* differentiation is not easy either because of the lack of many conditions observed *in vivo*. The aim of the present study was to improve the efficiency of rat ASC-derived cardiomyocytes to optimize their *in vitro* generation for further applications. ASCs were induced to cardiomyocyte lineage using a combination of two growth factors critically implicated in embryonic cardiomyogenesis (BMP-4 and VEGF) followed by a commercial methylcellulose-based medium with cytokines (IL-3 and IL-6), which had previously reported a cardiomyogenic potential.

## 2. Materials and Methods

### 2.1. Isolation and Maintenance of Cell Culture of Adipose Tissue-Derived Mesenchymal Stem Cells

ASCs were isolated with a combination of mechanical dissociation and collagenase incubation from Wistar rat subcutaneous adipose tissue, following previously reported protocols [24], adapted for rat tissue. Briefly, the rats were euthanized in a CO_2_ chamber accordingly with the institution's ethical guidelines for animal research procedures. With an antiseptic technique, rat adipose subcutaneous tissue was obtained and cut in small pieces until the tissue had an emulsion appearance. To digest the tissue, we used 0.1% collagenase type II (Sigma) and shaken-incubated at 37°C for 45 minutes. Then, collagenase activity was inactivated with Dulbecco's modified Eagle medium low glucose (DMEM-lg) (Gibco) supplemented with 10% fetal bovine serum (FBS) (Corning) and 1% antibiotic-antimycotic (Anti-Anti (100x),100 U/mL penicillin, 10 mg streptomycin, and 25 *μ*g amphotericin B per mL, Gibco). The inactivated digestion was passed through a sterile layer of chiffon. The remaining adipocytes and fats were discarded by centrifugation. The pellet stromal vascular fraction (SVF) was washed with DMEM-lg-supplemented medium and resuspended with 1x ACK lysis buffer to lyse red blood cells in the SVF. After 2 steps of centrifugation with DMEM-lg-supplemented medium, the cell pellets containing the ASCs were resuspended in DMEM-lg-supplemented medium, passed through a 100 *μ*m strainer, and then seeded in plates. The cells were evaluated every 24 hours with an inverted microscope, and the medium was changed every 3 days until cells have reached 70-80% confluence for cell passage.

### 2.2. Isolation of Rat Neonatal Cardiomyocytes

Rat neonatal cardiomyocytes were isolated from hearts of 1- to 4-day old neonate rats. The extracted hearts were shaken-incubated with trypsin 0.01% (trypsin type III from bovine pancreas) (Sigma) and Ham's F-10 media (Gibco) without serum until the tissue was disaggregated. Every 15 minutes, the supernatant was collected and inactivated with Ham's F-10 media with 20% FBS. The solution with disaggregated heart cells was centrifugated at 1,200 RPM for 5 minutes. The cell pellet was resuspended and seeded with Ham's F-10 media with calcium chloride 135 mg/mL, 20% FBS, and 1% antibiotic-antimycotic (Anti-Anti (100x),100 U/mL penicillin, 10 mg streptomycin, and 25 *μ*g amphotericin B per mL, Gibco). To obtain a pure cardiomyocyte culture, the seeded cells were incubated for 45-60 minutes at 37°C in a humid 5% CO_2_ incubator, and noncardiomyocyte cells adhere to the flask. After 45-60 minutes, the nonadherent cells (cardiomyocytes) were collected and seeded in a new flask. We observed contractile clusters 24 hours post-isolation of the rat neonatal cardiomyocyte cells. The cardiomyocytes were maintained in culture until being processed as a positive control for experiments.

### 2.3. Flow Cytometry

Characterization of rat ASC was performed by characteristic stem cell surface markers (CD90, RT1A, CD44, CD29, CD73, CD31, CD45, and CD34) using flow cytometry. Cell analysis was achieved in the platform BD FACSCalibur™ (Becton Dickinson, San Jose, CA) with CellQuest software (Becton Dickinson). Other surface markers related to cardiomyocyte differentiation such as CD106 (VCAM) and SIRP*α* (CD172a) were also evaluated. Cardiac troponin T (cTnT) was evaluated in undifferentiated ASCs and in a pool of rat neonatal cardiomyocytes. To quantify the efficiency of cardiomyocyte differentiation, the percentage of cTnT-positive cells was determined by flow cytometry. Cells were harvested from culture flasks or plates using trypsin 0.25% (Gibco) and centrifuged at 1,500 rpm for 5 minutes. The cell pellet was washed twice with 1 mL of 1x PBS 0.8% bovine albumin (BSA) and 0.02% sodium azide. Fixation and permeabilization were performed with an Intracellular Staining Kit (Invitrogen), following the protocol according to the specifications provided by the manufacturer. ASCs were washed in 1 mL of 1x PBS plus 0.8% BSA and 0.02% sodium azide. The primary antibodies were incubated for 30-45 min at room temperature; in some cases, primary antibodies were directly labeled with a fluorophore. In other cases, we used secondary antibodies coupled to fluorescein isothiocyanate (FITC), phycoerythrin (PE), or CFL 647 for 30-45 min at room temperature. An unstained control and a negative control (an appropriate isotype control) were used for each antibody. Antibodies used are listed in Supplementary Table 1.

### 2.4. Immunofluorescence Staining

The expressions of the stem cell marker CD90 and cardiac markers were analyzed in undifferentiated ASCs and in experimental condition cultures using immunofluorescence staining. The protocol used was as follows: cell cultures were washed 3 times with 1x PBS at 4°C; the cells were fixed with 4% paraformaldehyde for 30 minutes at 4°C. Each plate was washed three times with 1x PBS, and the cells were permeabilized with 0.5% Triton X-100 solution for 10 minutes and washed again 3 times with 1x PBS at 4°C. Then, they were incubated with a blocking solution (Biocare Medical, Background Sniper) for 10 minutes and washed 3 times with 1x PBS at 4°C. The primary antibodies were incubated for 45-60 minutes at room temperature (1 : 100-1 : 200). Cells were washed 3 times with 1x PBS at 4°C, and secondary antibodies were incubated (1 : 200-1 : 300) for 45 minutes at room temperature. Stained cell cultures were mounted with a mounting media containing 4′,6-diamidino-2-phenylindole (DAPI) (Santa Cruz ChemCruz ™ Ultracruz™ Mounting Medium). The images were obtained by confocal microscopy using a LSM 700 Zeiss microscope.

### 2.5. *In Vitro* Cardiomyocyte Differentiation from ASCs

**Figure 1:**
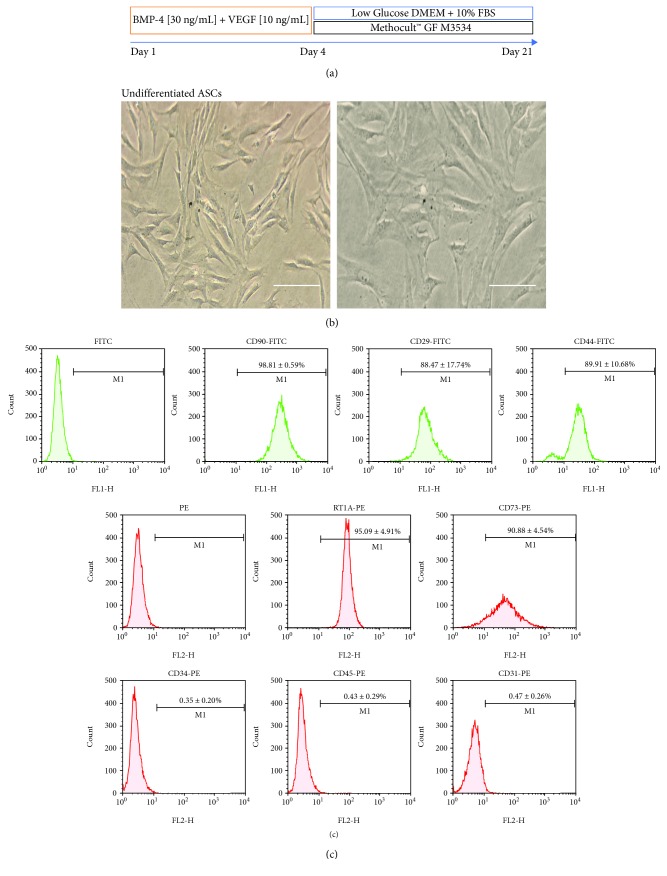
(a) Schematic outline of the directed cardiomyocyte differentiation protocol (days). Only growth factors and the combination of growth factors plus MethoCult M3534 conditions are shown. Undifferentiated ASCs. (b) A spindle fibroblast-like cell morphology was assessed by inverted microscope imaging (scale bar, 100 *μ*m). (c) Flow cytometry histograms for stem cell surface markers versus forward scatter (FSC), M1 (percentage of positive cells (%), mean ± SD, *n* = 3).

To optimize the terminal efficiency toward a cardiac lineage, we performed a directed cardiomyocyte differentiation protocol using a combination of important growth factors involved in the cardiac mesoderm specification and a methylcellulose-based medium with cytokines with a cardiomyogenic potential. For each biological replicate, the cells were stained with trypan blue and counted in a Neubauer chamber. Undifferentiated ASCs were seeded in a monolayer in 6-well plates (3 × 10^5^). ASCs were treated for 4 days with DMEM-lg medium supplemented with human BMP-4 [30 ng/mL] (ProSpec) and rat VEGF-C [10 ng/mL] (ProSpec). At day 5, the medium was changed by a commercial methylcellulose medium with cytokines (MethoCult™ GF M3534, STEMCELL Technologies) mainly containing Iscove's Modified Dulbecco's Medium (IMDM), 1% methylcellulose, fetal bovine serum, bovine serum albumin, recombinant human insulin, human transferrin (iron-saturated), 2-mercaptoethanol, recombinant mouse stem cell factor (SCF), recombinant mouse interleukin 3 (IL-3), recombinant human interleukin 6 (IL-6), and supplements (Figure 1(a)).

To assess reproducibility, we performed three biological replicates. Each experiment condition (untreated ASCs control, treated only with factors BMP-4 + VEGF) and the directed cardiomyocyte differentiation protocol (BMP-4 + VEGF followed by M3534) and each biological replicate were followed every day under a bright-light microscope. To determine the efficiency of cardiomyocyte differentiation, the expression of cardiac markers such as cardiac troponin T (cTnT), Gata-4, myosin heavy chain (MyH), and SerCa2 was analyzed, by flow cytometry and/or immunofluorescence.

### 2.6. Statistics

Data are presented as mean ± SD. Two-tailed Student's *t*-test was used to determine the statistical significance of differences between groups. A *p* value of <0.05 was considered statistically significant.

## 3. Results

### 3.1. Isolation and ASC Characterization

ASCs were obtained from subcutaneous rat adipose tissue by mechanical disaggregation and enzymatic digestion. The plated cells were monitored every 24 hours. During their proliferation, the isolated ASCs showed the distinctive spindle fibroblast-like morphology (Figure 1(b)). On passage 3 (*n* = 3), the cells were evaluated and characterized by flow cytometry and immunofluorescence to confirm that isolated ASCs maintain their phenotypic characteristics after growth in culture.

For characterization, the ASC immunophenotype was performed with previously established stem cell surface markers. It was observed that ASCs present characteristic stem cell surface markers. ASCs in the undifferentiated state were positive for CD90 (99.36 ± 0.59%), CD29 (88.47 ± 17.74%), CD44 (89.91 ± 10.68%), RT1A (96.91 ± 4.18%), and CD73 (97.75 ± 1.93%) and were negative for hematopoietic markers such as CD45 (0.53 ± 0.24%), CD34 (0.44 ± 0.11%), and the endothelial marker CD31 (0.47 ± 0.26%) (Figure 1(c)). Immunofluorescence observed by a confocal microscope showed the presence of CD90 in undifferentiated ASCs (Supplementary Figure 1).

### 3.2. Undifferentiated ASCs and Cardiomyocyte Markers

**Figure 2:**
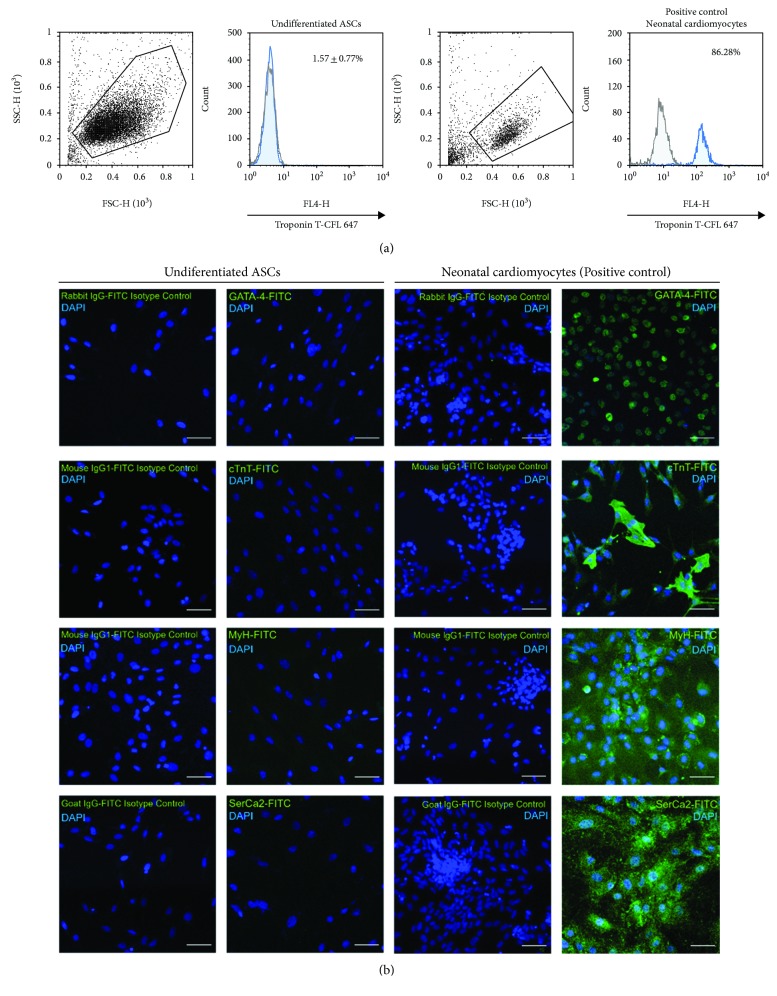
Undifferentiated ASCs do not express cTnT, a specific cardiomyocyte marker. Flow cytometry analysis of cTnT of undifferentiated ASCs. Histogram overlay showing isotype control goat IgG-CFL-647 (black line) and cTnT-CFL 647 (blue). Percentage of cTnT-positive cells by flow cytometry; results are shown as mean ± SD ASCs (*n* = 3). Flow cytometry analysis of cTnT was performed in a pool of neonatal cardiomyocytes (isolation of 10 neonatal rat hearts); histogram overlay showing isotype control goat IgG-CFL-647 (black line) and cTnT-CFL 647 (blue). Flow cytometry for cTnT. (b) Undifferentiated ASCs do not express specific cardiomyocyte markers. Undifferentiated ASC and rat neonatal cardiomyocyte (positive control) immunostaining for Gata-4, cTnT, MyH, and SerCa2, each one with its isotype control (negative control); image obtained by confocal microscopy (scale bars, 50 *μ*m).

To evaluate if ASCs (*n* = 3) in the undifferentiated state express cardiomyocyte markers, ASCs were analyzed for the presence of positive cells for the cTnT marker by flow cytometry; the cells do not express cTnT in the undifferentiated state (1.57 ± 0.77%) (Figure 2(a)). No positive cells were observed for cardiomyocyte markers such as Gata-4, cTnT, Myh, and SerCa2 by immunofluorescence (Figure 2(b)).

### 3.3. ASC Differentiation toward Cardiomyocytes with a Directed Cardiomyocyte Differentiation Protocol

To increase the terminal efficiency of cardiomyocyte differentiation from ASCs, we conducted an induction differentiation experiment using a combination of different culture conditions without previous culture selection. We induced cardiac differentiation from ASCs in three independent biological replicates, with a directed cardiomyocyte differentiation protocol that includes two critical cardiomyogenesis growth factors (BMP-4 [30 ng/mL] and VEGF [10 ng/mL]) and a methylcellulose-based medium with cytokines (MethoCult™ GF M3534) (Figure 1(a)).

**Figure 3:**
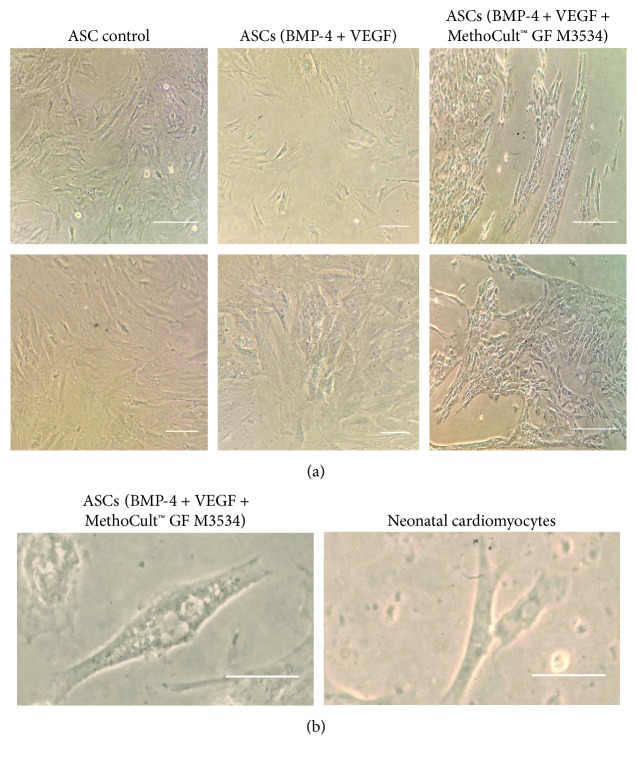
Cell morphology during cardiomyocyte differentiation from ASCs. (a) Control, only factors (BMP-4 + VEGF), and the directed cardiomyocyte differentiation protocol (MethoCult™ GF M3534 plus factors) conditions were observed by inverted microscope imaging (scale bar, 100 *μ*m). (b) Binucleated cardiomyocyte-like cells observed at day 10 in the directed cardiomyocyte differentiation protocol (MethoCult™ GF M3534 plus factors) conditions and an image of binucleated rat neonatal cardiomyocyte (scale bar, 100 *μ*m).

**Figure 4:**
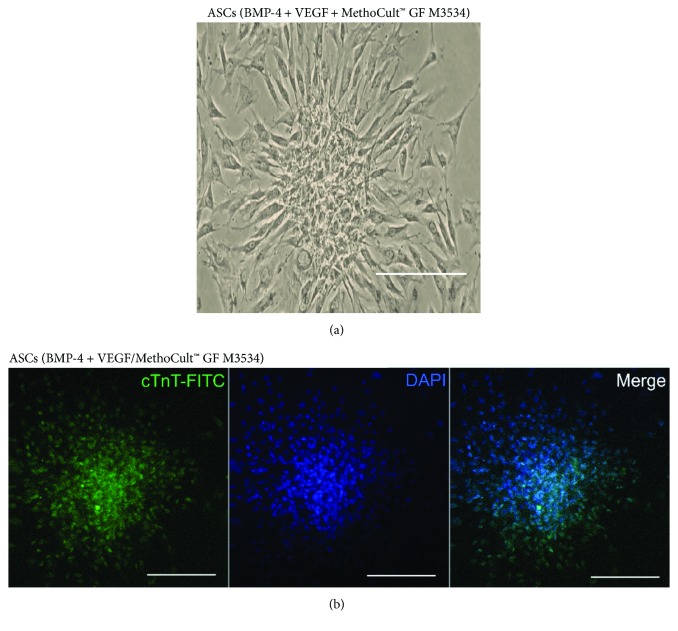
Cell cluster formation at day 15 in the directed cardiomyocyte differentiation protocol showing the “Rosette-like structures” that express cTnT. (a) Images obtained by inverted microscope imaging in conditions were observed by inverted microscope imaging (scale bar, 100 *μ*m). (b) Immunostaining for cTnT; image obtained by confocal microscopy (scale bars, 50 *μ*m).

From day 7, some cell morphology changes were observed during the cardiac induction experiments (Figure 3). Compared with only factors and nontreated control conditions, the changes were evident in cell cultures within the condition induced with the directed cardiomyocyte differentiation protocol. Cell cultures treated with the directed cardiomyocyte differentiation protocol showed a cell alignment pattern, elongated cells, dense cytoplasm with myofilament-like structures (Figure 3(a)), and some binucleated cells similar to cardiomyocytes (Figure 3(b)). Also, cell clusters were observed at day 15: these clusters were not observed in only factors and nontreated control conditions (Figure 4(a)). Despite that we observed morphologic changes compatible with cardiomyocyte characteristics, there were no spontaneous beating cells; however, we observed that these cell clusters express cTnT showed by immunofluorescence in the cultures treated with the factors plus MethoCult™ GF M3534 (Figure 4(b)).

**Figure 5:**
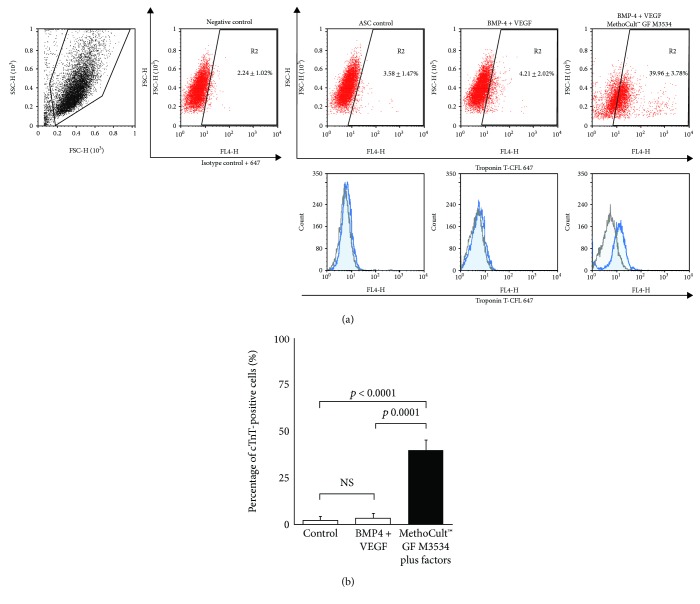
ASC-derived cardiomyocyte-like cells. (a) Flow cytometry for cTnT versus forward scatter (FSC) was performed at day 15. The scatter plot shows R2 (percentage of positive cells (%), mean ± SD). Histogram overlays showing isotype control goat IgG-CFL-647 (black line) and cTnT-CFL 647 (blue). (b) Percentage of cTnT-positive cells obtained by flow cytometry at day 15. Results are shown as mean ± SD of three independent experiments (*n* = 3). Each condition was compared (Student's *t*-test) against control and factors against the directed cardiomyocyte differentiation protocol (MethoCult™ GF M3534 plus factors).

To assess the efficiency of ASC-derived cardiac differentiation, we evaluated the percentage of cTnT-positive cells by flow cytometry at day 15. ASCs treated with the cardiomyocyte differentiation protocol obtained the highest percentages of cTnT-positive cells (39.96%±3.78, *p* < 0.0001 and *p* = 0.0001) in comparison with the induction with only factors (4.21 ± 2.02%) and control (3.58 ± 1.47%) conditions (Figures 5(a) and 5(b)).

**Figure 6:**
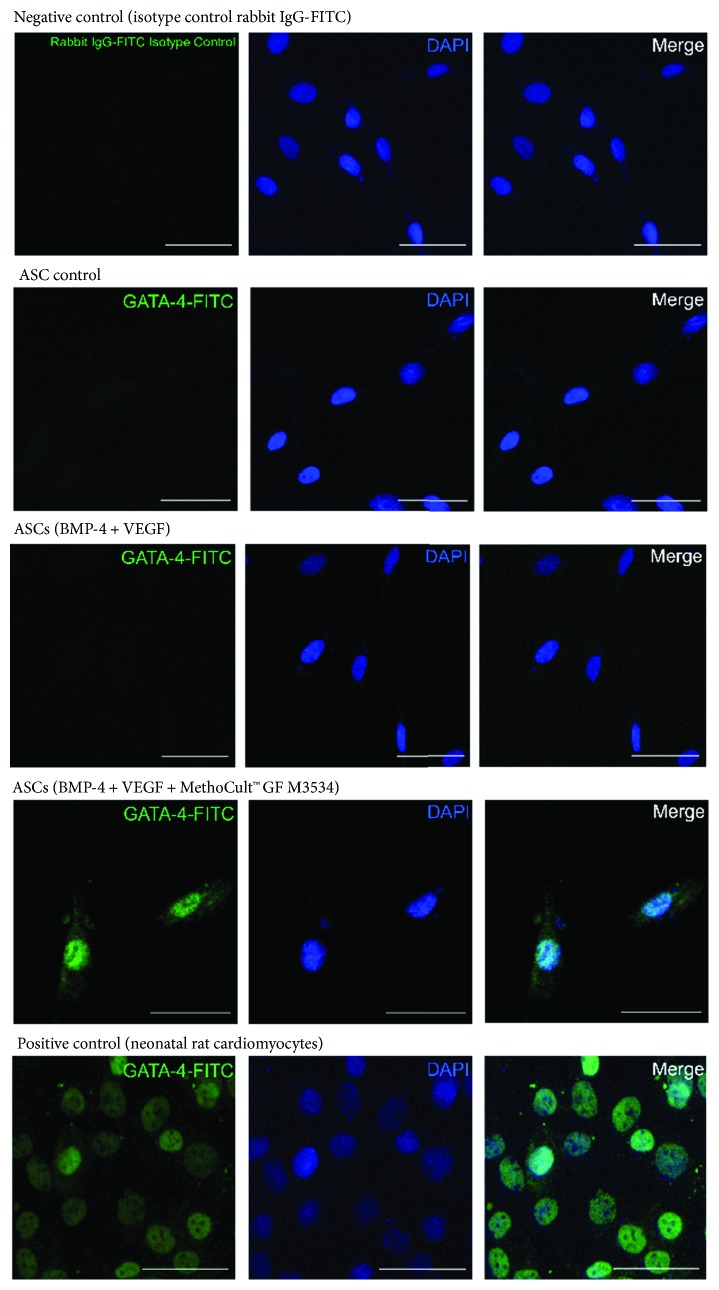
ASC-derived cardiomyocyte-like cells express cardiomyocyte markers. Immunostaining at day 21 for Gata-4 isotype control (negative control), ASC control, ASCs treated only with growth factors (BMP-4 and VEGF), and the directed cardiomyocyte differentiation protocol (MethoCult™ GF M3534 plus factors), and rat neonatal image cardiomyocyte, obtained by confocal microscopy (scale bars, 50 *μ*m).

**Figure 7:**
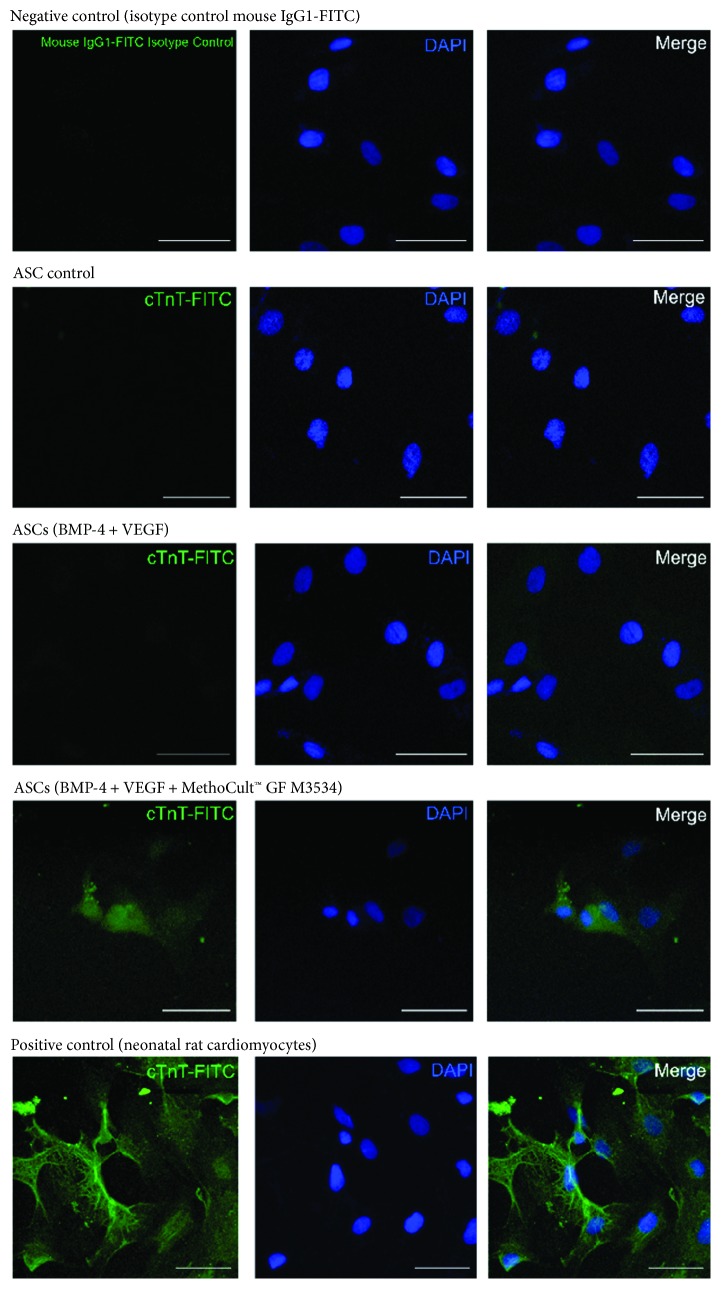
ASC-derived cardiomyocyte-like cells express cardiomyocyte markers. Immunostaining at day 21 for cTnT isotype control (negative control), ASC control, ASCs treated only with growth factors (BMP-4 and VEGF), and the directed cardiomyocyte differentiation protocol (MethoCult™ GF M3534 plus factors), and rat neonatal image cardiomyocyte, obtained by confocal microscopy (scale bars, 50 *μ*m).

**Figure 8:**
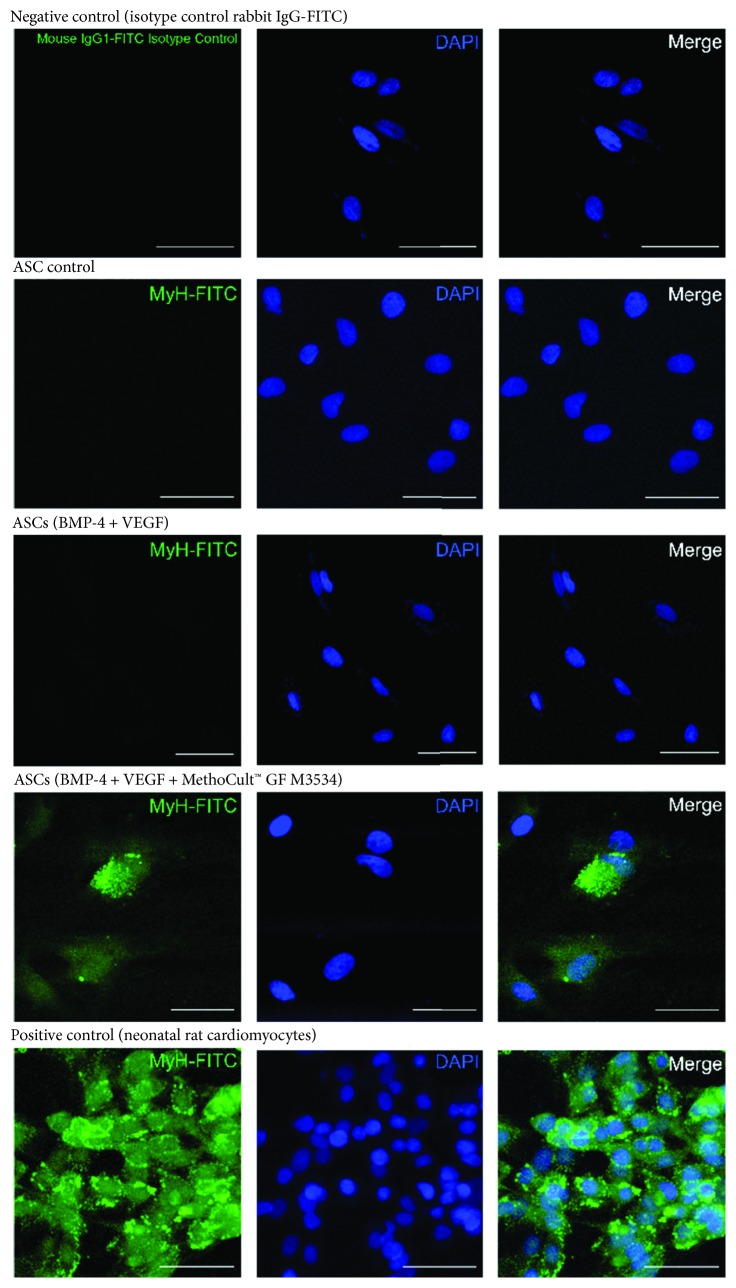
ASC-derived cardiomyocyte-like cells express cardiomyocyte markers. Immunostaining at day 21 for MyH. Isotype control (negative control), ASC control, ASCs treated only with growth factors (BMP-4 and VEGF), and the directed cardiomyocyte differentiation protocol (MethoCult™ GF M3534 plus factors), and rat neonatal image cardiomyocyte, obtained by confocal microscopy (Scale bars, 50 *μ*m).

**Figure 9:**
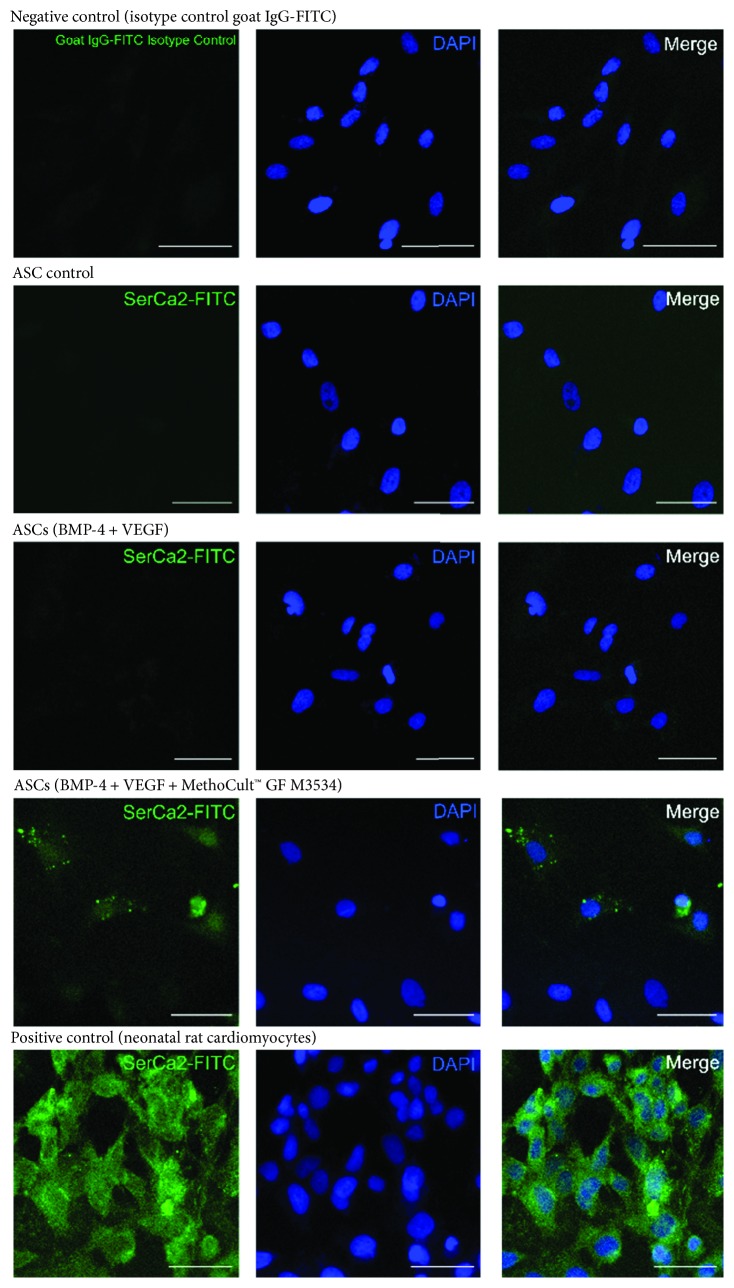
ASC-derived cardiomyocyte-like cells express cardiomyocyte markers. Immunostaining at day 21 for SerCa2. Isotype control (negative control), ASC control, ASCs treated only with growth factors (BMP-4 and VEGF), and the directed cardiomyocyte differentiation protocol (MethoCult™ GF M3534 plus factors), and rat neonatal image cardiomyocyte, obtained by confocal microscopy (scale bars, 50 *μ*m).

On day 21, further characterization was performed in ASC-derived cardiac-like cells. It was observed, by immunofluorescence, that ASCs induced with the directed cardiomyocyte differentiation protocol express specific cardiomyocyte markers such as Gata-4 (Figure 6), Troponin T (cTnT) (Figure 7), Myh (Figure 8), and SerCa2 (Figure 9), but their expression was lower compared with the rat neonatal cardiomyocytes.

### 3.4. CD106 and SIRP*α* Characterization in Undifferentiated ASCs

**Figure 10:**
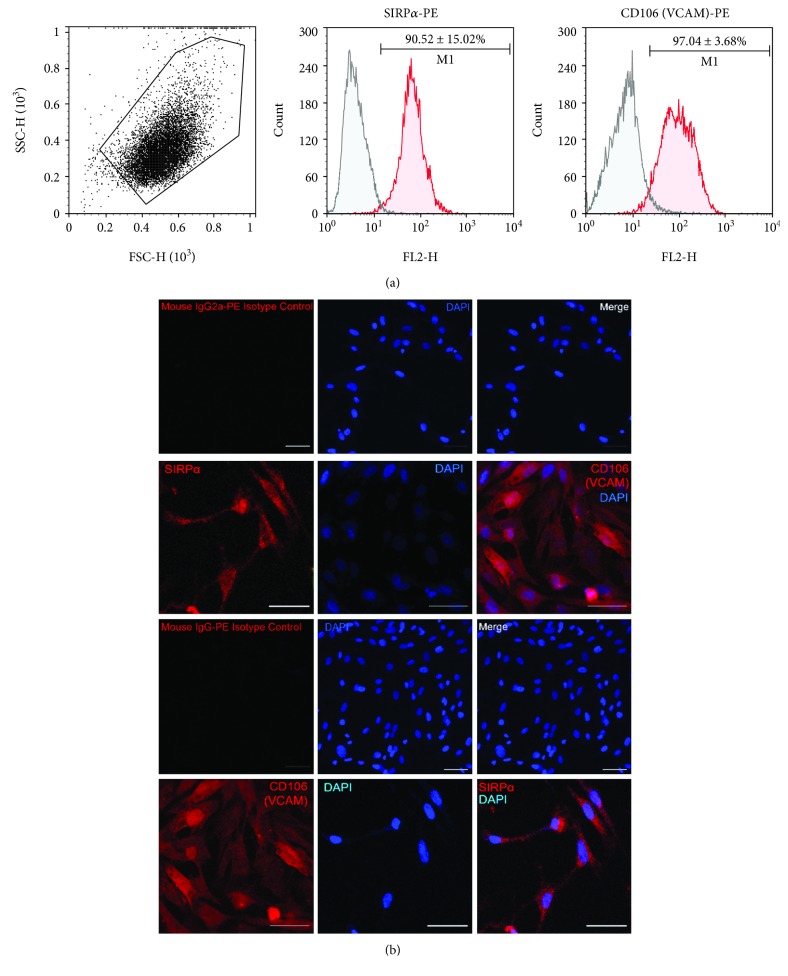
Undifferentiated ASCs highly express CD106 and SIRP*α*. (a) Flow cytometry histogram overlays, isotype control (black line) and CD106 or SIRP*α* (red), and percentage of CD106 and SIRP*α* positive cells obtained by flow cytometry; results are shown as mean ± SD (*n* = 3). (b) Immunostaining for CD106, SIRP*α*, and isotype controls; image obtained by confocal microscopy (scale bars, 50 *μ*m).

Expressions of CD106 (VCAM) and SIRP*α* (CD172a) have been reported to be useful for IPSC-derived cardiomyocyte enrichment and separation with cell sorting. We characterized undifferentiated ASCs with these two cardiomyocyte surface markers to know if it was possible to use these markers for an enrichment step after ASC-derived cardiomyocyte experiments. Both markers showed a high percentage of positive cells (Figure 10(a)), CD106 (97.04 ± 3.68%), and SIRP*α* (90.52 ± 15.02%). Also, as it was observed by immunofluorescence, undifferentiated ASCs were highly expressed, limiting its use for an enrichment step after ASC cardiomyocyte induction with the directed cardiomyocyte differentiation protocol (Figure 10(b)).

## 4. Discussion

The ability to differentiate into a cardiogenic lineage has been studied in different kinds of stem cells. The most efficient protocol was reported in IPSC-derived cardiomyocytes with a high differentiation efficiency, but until now their uses in clinical applications have been diminished by its potentially tumorigenic formation and some other limitations [2, 12]. ASCs are suitable for clinical applications with some advantages over other types of stem cells. ASC-derived cardiomyocytes are an alternative; however, the protocols so far have shown a low and variable *in vitro* cardiomyocyte differentiation; besides, they do not quantify the differentiation efficiency with a quantitative method.

Here, we performed a novel directed cardiomyocyte differentiation protocol from rat ASCs that combined the use of critical cardiomyogenic growth factors, such as BMP-4 [25] and VEGF [26, 27], in the first 4 days of the experiment to commit undifferentiated ASCs to cardiac progenitor cells, followed by a semi-solid commercial medium based on methylcellulose and cytokines (IL-3 and IL-6) that had previously been reported with cardiomyogenic potential [6, 7, 23, 28], to further cardiomyocyte differentiation.

Our novel approach improved the *in vitro* differentiation of rat ASCs toward cardiomyocyte-like cells in up to 44.03%, showing a high efficiency and reproducibility without a selection step for cardiomyocyte enrichment. The induction with the directed cardiomyocyte differentiation protocol significantly increased the differentiation efficiency toward an immature cardiomyocyte with a positive expression of early (Gata-4) and late cardiac differentiation markers (cTnT and Myh). Moreover, we also observed cardiomyocyte maturation features with the expression at day 21 of SERCa2.

ASC-derived cardiomyocyte protocol performed in the present study was assessed in three independent biological replicates, evaluating quantitatively the cardiomyocyte differentiation efficiency. The cardiomyocyte differentiation efficiency was measured with a quantitative tool. This is important because it is the only way to compare protocols and to know the number of cells that are useful for further applications. Flow cytometry is a reliable way to assess it because it is a high-sensitivity analytical tool [22]. So far, only two studies and ours quantify the cardiomyocyte differentiation efficiency by flow cytometry [7, 16]. The great majority of ASC-derived cardiomyocyte protocols count the number of beating clones or the percentage of positive expressing cells for cardiac markers in a qualitative manner [6, 14, 17–19, 23, 29–31].

The proposed cardiomyocyte differentiation directed protocol in the present study improves the efficiency to obtain ASC-derived cardiac-like cells without any selection steps. Léobon and collaborators [7] propose a three-step process to improve cardiomyocyte differentiation efficiency from ASCs: (1) seed the ASCs in a methylcellulose medium until the cell clusters appear, (2) dissect under an inverted microscope to recollect manually the cell clusters, and (3) seed the recovered clusters in a BHK-21 liquid medium, obtaining two types of populations (nonadherent and adherent cells) approximately with 50% cTnT-positive cells each one [7]. If the selection step was not assessed, the percentage of cTnT-positive cells was reduced to 10% [7]. Our cardiac differentiation protocol showed similarly high differentiation efficiency as the previously reported study [7], with a high percentage of cTnT-positive cells and the advantage of skipping the pick-up selection step for cardiomyocyte enrichment.

Compatible cardiomyocyte morphological changes were observed during the cell culture monitoring, such as elongated, bifurcated, and binucleated cells. The presence of a cellular alignment and the development of cell clusters resembling rosette-like structures caught our attention. The formation of these structures has been linked as an essential process in morphogenesis, both *in vitro* and *in vivo* [32]. Rosette-like structures observed in cultures treated with the directed cardiomyocyte differentiation protocols support the cardiomyocyte development from undifferentiated ASCs.

Unlike previous studies, no spontaneous beating that would allow us to perform a functional analysis was observed. Spontaneous beating or automatism in an *in vivo* environment is a characteristic of early fetal cardiomyocytes; as the development occurs, the cells become more specialized allowing the intervention of the nodal system, losing their automaticity as cells maturate. Pluripotent stem cell-derived cardiomyocytes show a significantly reduced SERCa2 expression, and only in the long term could cultures overcome its expression and enhance cardiomyocyte maturation [2]. Despite not having observed spontaneous beating cells in our differentiation protocol, the positive expression of a sarcoplasmic/endoplasmic reticulum Ca^2+^-ATPase (SERCa2) implicated in the control of calcium transportation in the cardiomyocyte endoplasmic reticulum shows a further cardiomyocyte maturation [33], which could explain in some way the lack of automatism in ASC-derived adult cardiomyocyte-like cells.

No surface marker capable of separating the ASC-derived cardiomyocytes has been reported before. So far, almost all pluripotent stem cell-derived cardiomyocytes with high efficiency include a cardiomyocyte purification step to enhance the differentiation yield [11, 34–37]. In an attempt to obtain a purer population and a higher efficiency of ASC-derived cardiomyocytes-like cells, we considered to enrich by fluorescence-activated cell sorting using two IPSC-derived cardiomyocyte surface markers previously reported, CD106 (VCAM) and SIRP*α* (CD172a) [38, 39]; unfortunately, both were highly expressed in the undifferentiated ASCs making difficult its use for enrichment of the differentiated ASC-derived cardiac-like cells.

Cardiomyocyte development *in vitro* has a high grade of complexity; therefore, differentiating cells with a single small molecule or single growth factor is insufficient to induce cardiomyogenesis in ASCs. Cardiomyogenesis is an extremely complex process that depends on the interaction of different signaling pathways, in a time-, space-, and dose-dependent manner. To date, the better and high-efficiency stem cell differentiation protocols require the combination of several small molecules and growth factors, cocultures, and even three-dimensional structures, mimicking the embryonic cardiac development [1].

Not surprisingly, the directed cardiomyocyte differentiation protocol presented here that resembles the cardiac embryonic development with cardiac-specific growth factors and cardiogenic cytokines breaks the paradigm that undifferentiated ASCs should be treated differently as pluripotent stem cells in matters of cardiomyocyte differentiation. Stimulation with growth factors or small molecules critically involved in cardiomyogenesis was also evaluated in other protocols, such as the use of TGF-*β*1 [16] or Wnt noncanonical antagonist (Wnt5a + Dkk1) [23]. Both studies result in an induction of cardiac-like cells of cultured ASCs. Moreover, some undifferentiated ASCs have been reported with a positive expression of pluripotent markers, such as Oct3/4, Sox2, Nanog, SSEA-3, and SSEA-4 [40–42], supporting this hypothesis.

Despite the high efficiency observed to obtain cardiomyocyte-like cells with the presented method, more research is needed to optimize differentiation toward a more mature and functional cardiomyocyte for modeling-disease or pathophysiological applications. However, probably induced ASCs toward a cardiac lineage with the present directed cardiomyocyte differentiation protocol could be transplanted in an *in vivo* environment to continue the maturation process and, at the same time, could amend the infarction myocardium to a better prognosis [18, 43].

It is crucial to look for an appropriate ASC-derived cardiomyocyte-like cell cardiac surface marker as it would be advantageous for an enrichment step that could increment considerably the differentiation efficiency. The high cost of the growth factors can limit the present protocol for scale-up purposes; substituting them with interchangeable small molecules implicated in the cardiogenic signaling pathways could lower the cost. This novel and easy method could be useful as a basis for further research in cardiomyocyte differentiation from ASCs. Evaluation of the present protocol in human ASCs is very important: once efficiency in human cells is proven, this method could be implemented in different applications, such as disease modeling, cell therapy, and drug discovery.

## 5. Conclusions

The ASC-derived cardiomyocyte is a suitable alternative for clinical applications, but cardiomyocyte development *in vitro* has a high grade of complexity. For that reason, it is important to search and describe the most appropriate protocol to ensure optimal results. The combination of BMP-4 and VEGF plus a semisolid methylcellulose-based medium containing IL-3 and IL-6 appears to be a good inducer of cardiac differentiation in the present protocol with a high differentiation efficiency. It is a challenge to identify the best combinatorial, but this is an example that by stimulating the pathways involved in embryonic cardiomyogenesis, we can increase the efficiency of cardiomyocyte differentiation derived from ASCs. Clinical applications will be possible for a long term and with further research.

## Data Availability

Access to the datasets used to support the findings of this study will be considered by the author upon request.

## Supplementary Materials

Supplementary Materials Supplementary Table 1: list of primary and secondary antibodies used for flow cytometry and immunofluorescence. Supplementary Figure 1: undifferentiated ASC immunostaining for CD90, image obtained by confocal microscopy (scale bars, 50 *μ*m). Supplementary Figure 2: bar plots containing the mean fluorescence intensity of the immunofluorescence images for different cardiac markers in ASCs control, BMP4 + VEGF, BMP-4 + VEGF plus MethoCult™ GF M3534, and rat neonatal cardiomyocytes. Results are shown as the mean ± SD. MFI was analyzed with ZEN 2.5 Blue edition software, Carl Zeiss AG.
