# Supplementary material for: Improved Efficiency of Cardiomyocyte-Like Cell Differentiation from Rat Adipose Tissue-Derived Mesenchymal Stem Cells with a Directed Differentiation Protocol
Source: Stem Cells Int. 2019 Apr 1;2019:8940365. doi: 10.1155/2019/8940365 (PMC6466858; doi:10.1155/2019/8940365)
Supplement: Supplementary Materials — Supplementary Table 1: list of primary and secondary antibodies used for flow cytometry and immunofluorescence. Supplementary Figure 1: undifferentiated ASC immunostaining for CD90, image obtained by confocal microscopy (scale bars, 50 μm). Supplementary Figure 2: bar plots containing the mean fluorescence intensity of the immunofluorescence images for different cardiac markers in ASCs control, BMP4 + VEGF, BMP-4 + VEGF plus MethoCult™ GF M3534, and rat neonatal cardiomyocytes. Results are shown as the mean ± SD. MFI was analyzed with ZEN 2.5 Blue edition software, Carl Zeiss AG. [file 8940365.f1.pdf]

Supplementary Table 1. List of primary and secondary antibodies.

Antibody	Clone	Catalog Number	Brand
**Primary antibodies**			
PE anti-rat RT1A (MHC Class I)	OX-18	205208	BioLegend®
PE anti-rat CD45 (LCA)	OX-1	202207	BioLegend®
PE anti-rat CD106 (VCAM-1)	MR106	200403	BioLegend®
PE anti-rat CD172a (SIRPα)	OX-41	204706	BioLegend®
FITC anti-mouse/rat CD90.1 (Thy-1.1)	HIS51	11-0900-85	eBioscience™
FITC anti-rat CD44H (Pgp-1, H-CAM)	OX-49	203906	BioLegend®
FITC anti-mouse/rat CD29 (integrin β1)	HMβ1-1	102205	BioLegend®
CD73 anti-Rat	5F/B9 (RUO)	551123	BD Pharmingen™
CD34 Antibody	MEC 14.7	sc-18917	Santa Cruz ®
CD31/PECAM-1 Antibody	TLD-3A12	NB100-64796	Novus Biological®
Troponin T-C Antibody	C-19	sc-8121	Santa Cruz ®
Cardiac Troponin T Antibody	13-11	MA5-12960	Invitrogen ™
SERCA2 Antibody	N-19	sc-8095	Santa Cruz ®
GATA-4 Antibody	H-112	sc-9053	Santa Cruz ®
MYH Antibody	TH81	sc101334	Santa Cruz ®
**Secondary antibodies**			
Donkey anti-goat IgG-CFL 647		sc362285	Santa Cruz ®
Donkey anti-rabbit IgG-FITC		406403	BioLegend ®
Rabbit anti-goat IgG-FITC		sc-2777	Santa Cruz ®
Goat anti-mouse IgG FITC		405305	BioLegend®
Goat anti-mouse IgG-PE		sc-3798	Santa Cruz ®
**Isotype Controls**			
Mouse IgG1, κ Isotype Ctrl Antibody	MOPC-21	400101	BioLegend®
PE Mouse IgG2a, κ Isotype Ctrl Antibody	MOPC-173	400211	BioLegend®
PE Mouse IgG1, κ Isotype Ctrl Antibody	MOPC-21	400111	BioLegend®
Rabbit IgG, Isotype control	Polyclonal	NBP2-24891	Novus Biological®
Goat IgG, Isotype control	Polyclonal	NB410-28088	Novus Biological®

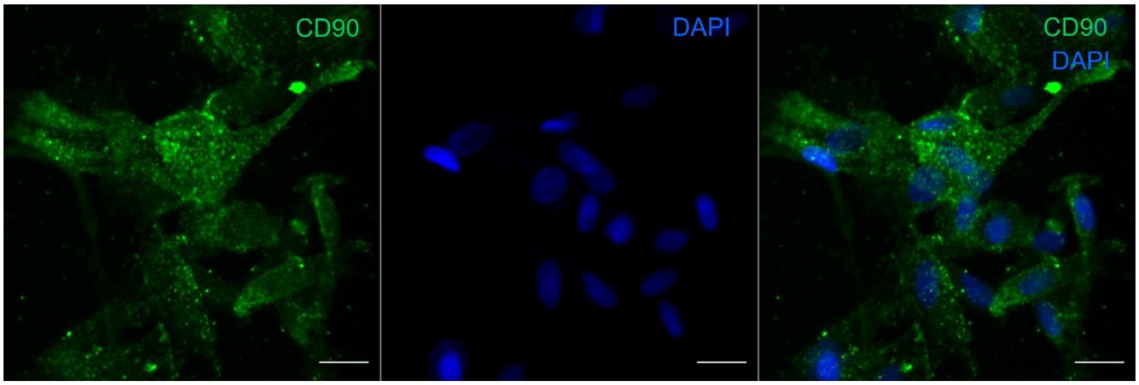

Supplementary Figure 1. Undifferentiated ASCs immunostaining for CD90, image obtained by confocal microscopy (Scale bars, 50 μm).

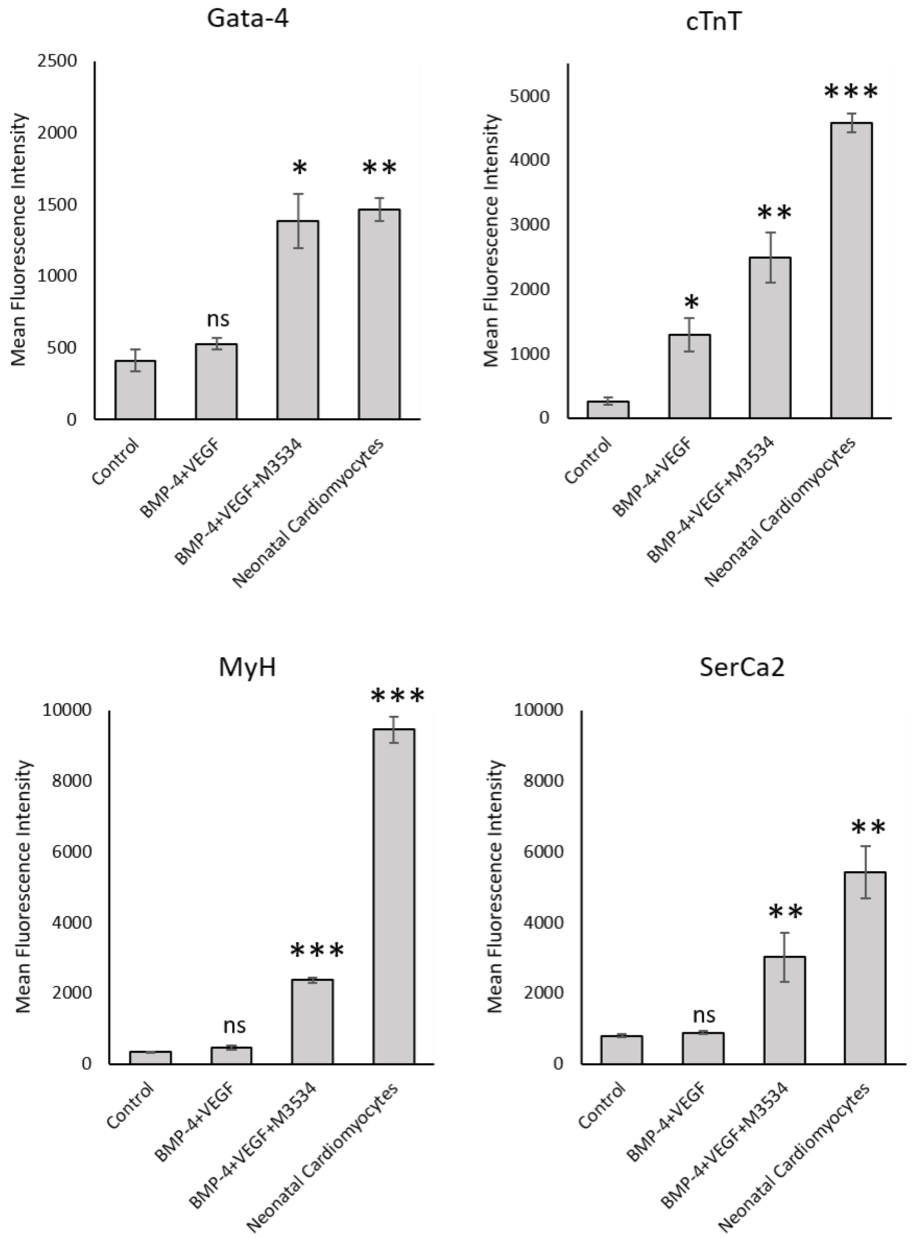

Supplementary Figure 2. Bar plots containing the mean fluorescence intensity (MFI) of the immunofluorescence images for different cardiac markers in ASCs control, BMP-4 + VEGF, BMP-4 + VEGF plus Methocult™ GF M3534, and rat neonatal cardiomyocytes. Results are shown as the mean ± SD. ns= No significant, *p<0.05, ** p<0.01, *** p<0.001, each condition was compared (Student's t-test) against control. MFI was analyzed with ZEN 2.5 Blue edition software, Carl Zeiss AG.
